# Extended opening hours of the one-stop shop

**DOI:** 10.1186/bcr2994

**Published:** 2011-11-04

**Authors:** S Flais, M Dumba, A Newland

**Affiliations:** 1 Ealing Hospital, London, UK

## Introduction

The one-stop model was introduced into the oncology follow-up clinic for breast cancer patients at Ealing Hospital in November 2010. We present why and how this change was introduced, and assess the impact on radiology practice during the first 6 months. We also present the results of our first patient satisfaction survey completed by those attending the new clinic.

## Methods

A customer survey was completed by 50 patients attending the new follow-up clinic over a 2-month period. The impact on radiology practice was also assessed

## Results

A total 100% of patients preferred the new follow-up system, and organisational changes to the work flow were implemented with no significant additional cost.

## Conclusion

The one-stop approach is preferred by breast cancer patients at all stages of their care, not just at diagnosis. Patients can benefit from this system with minimal additional cost burden.

